# A novel locus on chromosome 1 underlies the evolution of a melanic plumage polymorphism in a wild songbird

**DOI:** 10.1098/rsos.160805

**Published:** 2017-02-15

**Authors:** Yann X. C. Bourgeois, Boris Delahaie, Mathieu Gautier, Emeline Lhuillier, Pierre-Jean G. Malé, Joris A. M. Bertrand, Josselin Cornuault, Kazumasa Wakamatsu, Olivier Bouchez, Claire Mould, Jade Bruxaux, Hélène Holota, Borja Milá, Christophe Thébaud

**Affiliations:** 1 Laboratoire Évolution et Diversité Biologique, UMR5174 CNRS, Université Paul Sabatier – ENFA, 31062 Toulouse Cedex 9, France; 2 INRA, UMR 1062 CBGP (INRA, IRD, Cirad, Montpellier SupAgro), Campus de Baillarguet, 34988 Montferrier-sur-Lez, France; 3 INRA, GeT-PlaGe, Genotoul, 24 chemin de Borde Rouge, Auzeville, CS 52627, 31326 Castanet-Tolosan, France; 4 INRA, UAR1209, 24 chemin de Borde Rouge, Auzeville, CS 52627, 31326 Castanet-Tolosan, France; 5 Department of Chemistry, Fujita Health University, School of Health Sciences, Toyoake Aichi 470-1192, Japan; 6 GenPhySE, Université de Toulouse, INRA, INPT, INP-ENVT, 24 chemin de Borde Rouge, Auzeville, CS 52627, 31326 Castanet-Tolosan, France; 7 National Museum of Natural Sciences, Spanish National Research Council (CSIC), 28006 Madrid, Spain

**Keywords:** melanism, chromosome 1, polymorphism, selective sweep, *Zosterops*

## Abstract

Understanding the mechanisms responsible for phenotypic diversification within and among species ultimately rests with linking naturally occurring mutations to functionally and ecologically significant traits. Colour polymorphisms are of great interest in this context because discrete colour patterns within a population are often controlled by just a few genes in a common environment. We investigated how and why phenotypic diversity arose and persists in the *Zosterops borbonicus* white-eye of Reunion (Mascarene archipelago), a colour polymorphic songbird in which all highland populations contain individuals belonging to either a brown or a grey plumage morph. Using extensive phenotypic and genomic data, we demonstrate that this melanin-based colour polymorphism is controlled by a single locus on chromosome 1 with two large-effect alleles, which was not previously described as affecting hair or feather colour. Differences between colour morphs appear to rely upon complex *cis*-regulatory variation that either prevents the synthesis of pheomelanin in grey feathers, or increases its production in brown ones. We used coalescent analyses to show that, from a ‘brown’ ancestral population, the dominant ‘grey’ allele spread quickly once it arose from a new mutation. Since colour morphs are always found in mixture, this implies that the selected allele does not go to fixation, but instead reaches an intermediate frequency, as would be expected under balancing selection.

## 1. Introduction

Identifying the specific genes underlying inter-individual phenotypic variation and reconstructing their evolutionary history is a key issue to link naturally occurring mutations to ecologically significant traits, and help resolve questions relative to the origin and maintenance of genetic and phenotypic variability in natural populations [1]. In this context, colour polymorphisms have played an essential role by enhancing our understanding of how selection and demography can impact phenotypes [2–5], and by identifying proximate causes of phenotypic variation [6,7]. Melanins, in contrast to other pigments such as carotenoids, allow the production of colour traits that in most cases are independent of the environment and the individuals' phenotypic condition [8,9] (but see also [10,11]) and show heritable segregation among colour morphs [12], a feature that appears to be critical for explaining the origin and maintenance of distinct colour morphs in natural populations [13]. Moreover, the observed covariation between melanin-based pigmentation and life-history or social strategies [14,15] suggests a major role for intergenic interactions and pleiotropic effects in the evolution of colour polymorphisms [16].

A great deal of research over the last decade has been devoted to elucidating the molecular basis of melanin-based colour polymorphism in natural populations. Early studies have assessed the role of the melanocortin-1-receptor (*MC1R*) gene, currently one of the most studied ‘colour genes’. This gene displays relatively low pleiotropic effect (but see [17]) and has been shown to explain colour variation in a broad variety of independent lineages, such as lizards [18], mice [19], humans [20], mammoths [21] and birds [22–25]. Although this suggested at first a relatively simple and general genetic mechanism underlying melanin-based polymorphisms, there is mounting evidence suggesting that mechanisms of hair/feather colour evolution may involve many other genes, and this seems particularly likely in species or populations in which the polymorphism arises from complex patterns of eumelanin/phaeomelanin deposition [26,27]. Studies in model species such as laboratory mice have shown that several genes other than *MC1R* could play a role in explaining differences in the patterning of melanin pigments across the body [28], and alternative candidate loci for melanic colour polymorphisms have indeed been identified in wild non-model species [29–32].

**Figure 1.**
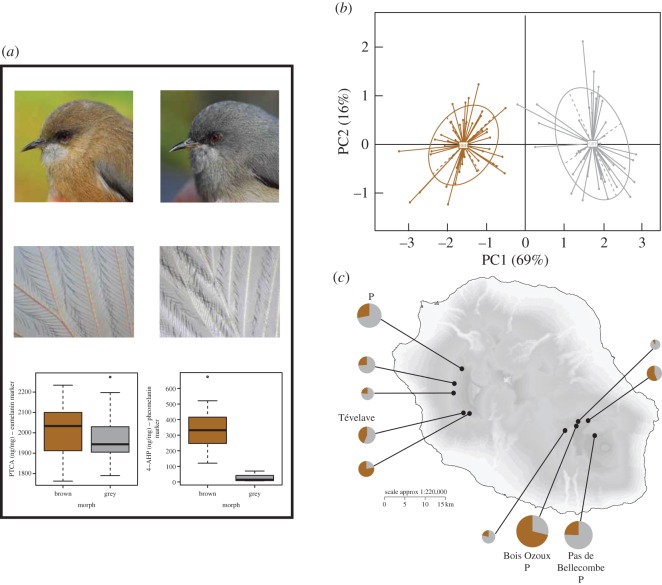
Phenotypic variation in highland populations of the Reunion grey white-eye and the population sampling scheme. (*a*) Photographs of brown and grey morphs (i), microscopic view of grey and brown feathers (ii) and melanin content per morph (iii) for 12 brown and 12 grey individuals. (*b*) PCA on spectrophotometry measures for feathers sampled from the back of brown and grey birds. (*c*) Morph frequencies across different localities. ‘P’ stands for localities included in pedigree analyses. Localities used for GBS and RAD-seq analyses are labelled. The size of the pie charts is proportional to sample size (ranging from 11 to 111 birds).

The recent development of next-generation sequencing has allowed research to transcend candidate-gene approaches in wild non-model species, leading to the identification of genomic regions associated with fitness-related traits, including colour traits [33,34]. Here, we took advantage of this technology and used a population genomic approach to investigate the genetic architecture underpinning a melanic plumage polymorphism in natural populations of the Reunion grey white-eye, *Zosterops borbonicus*, a textbook example of intraspecific variation in plumage coloration in birds [35]. This species displays four geographically structured plumage forms distributed across the small oceanic island of Reunion (2512 km^2^) [36]. One of these forms, restricted to the highlands of Reunion, comprises two distinct and sympatric colour morphs, with birds showing predominantly grey or brown plumage, respectively (figure 1). Melanic plumage polymorphism is widely maintained across the range of this highland form, with both grey and brown birds always present at any given locality, although populations vary in morph frequencies [30]. Field observations suggesting that mating between grey individuals could produce both grey and brown offspring, and that mating between brown individuals always produced brown offspring, led to the proposition that differences between grey and brown morphs could be due to genetic changes at a few loci of major effect [30].

Previously, we used a candidate-gene approach to search for associations between plumage colour and genetic variants in the Reunion grey white-eye, and did not detect any for a series of genes that play a critical role in melanin-based pigmentation patterns in other species, including *MC1R*, *Agouti*, *Corin*, *Pro-opiomelanocortin* and *Tyrosinase-related protein 1* [37,38]. In this study, we first confirmed that segregating phenotypes conform to a Mendelian model using genetic marker-based pedigree reconstruction (table 1). Then, using restriction site-associated DNA (RAD)-sequencing [41] on pools of individuals from three geographically separate populations [40], we performed a genome-wide scan relative to colour morphs while accounting for population structure. This approach provides a cost-effective way to detect associations by allowing large numbers of individuals to be included into a sample and by providing a sufficient density of markers for mapping (see table 1 for more details on the approaches used in this study). This led us to discover a single narrow region on chromosome 1 showing a strong association with melanin-based plumage colour phenotypes. We confirmed the existence of this association by using individual genotyping-by-sequencing (GBS) [42] and whole-genome sequencing of individuals with known pedigrees, and assessed the functional consequences of the molecular variants underlying plumage colour variation. Finally, we addressed the origin of the polymorphism by estimating the relative age of the genetic variants and tested the role of selection in explaining how the polymorphism could have spread and been widely maintained across populations.

**Table 1.** Summary of datasets used in this study.

method used	goal	notes	sample size	references
microsatellites	pedigree analysis to characterize inheritance patterns of plumage colour	obtained from two populations monitored since 2008 (Pas de Bellecombe and Bois Ozoux) and one population sampled in 2007 and 2012 (figure 1)	261	[39]
pooled RAD-sequencing	mapping loci associated with plumage colour	high density of markers (more than 600 000)	137 (6 pools)	[40]
GBS	validating associations obtained from pooled data with individual genotyping	lower density of markers than RAD-seq (25 000)	42	this study
whole-genome resequencing	annotating coding/non-coding mutations; characterizing selection in the genomic region associated with plumage colour	near-exhaustive coverage of the region associated with plumage colour	12 (6 parents and 6 offspring)	this study

## 2. Material and methods

### 2.1. Field sampling

Birds were captured in the field using mist nets between 2007 and 2012 on Reunion (55°39′ E; 21°00′ S), were weighed and marked with a uniquely numbered aluminium ring, and approximately 10 µl of blood was collected from each bird. Blood was conserved in Queen's lysis buffer [43] and stored at −20°C for long-term preservation. Individuals were sexed by PCR [44] in order to infer the number of distinct Z chromosomes included in each pool. On each bird, 10 feathers from four different body parts (head, back, flank and belly) were collected for spectrophotometric analyses. We also measured five morphological traits with a dial calliper (to the nearest 0.1 mm): tail length (from the uropygial gland to the tip of the longest rectrix), tarsus length (from the intertarsal joint to the most distal undivided scute on the tarsometatarsus), bill length (from the anterior end of the nares to the tip of the upper mandible), bill width and depth (both measured at the anterior end of the nares).

### 2.2. Measurement of the reflectance spectra of plumage patches

Ninety-six individuals from the 137 used for pooled RAD-sequencing were used for characterizing phenotypes by spectrophotometry analyses. We used reflectance spectrophotometry to characterize variation in plumage colour, because it provides an objective quantification of colour [45,46]. We summarized colour variation by conducting a principal components analysis (PCA) on a set of five variables summarizing the spectrum for back feathers that were then used for estimating melanin content. We determined whether birds were actually able to discriminate morphs by using Vorobyev & Osorio's model of colour perception [47]. This model allows chromatic contrasts between two colours to be compared with a threshold value of colour discrimination by birds (for further details of how this was perfomed, see [48]).

### 2.3. Melanin content

We used microanalytical methods to quantify the eumelanin and pheomelanin content of the feathers from brown and grey morphs (see [49] for a detailed description of the method). Feather samples (*ca* 7 mg) from the back of 12 brown and 12 grey individuals were homogenized with a Ten-Broeck homogenizer at a concentration of 10 mg ml^−1^ and 100 µl aliquots were subjected to Soluene-350 solubilization [50], alkaline hydrogen peroxide oxidation [51] and hydriodic acid hydrolysis [52]. Values are from single determinations. A500/mg and A650/mg values were substracted by background values of 0.019 and 0.001.

### 2.4. Pedigree analysis

Performing controlled matings in wild populations is challenging, and hence to study inheritance patterns, we inferred pedigrees in three natural populations monitored over multiple years at three localities. Given the reduced dispersal in *Z. borbonicus* [53,54], samples from these populations were more likely to include parents and their offspring. Pedigree reconstruction was performed using the Bayesian parentage assignment algorithm implemented in the R package MasterBayes v. 2.50 [55] and genotype data from an informative panel of 11 neutral microsatellites [39]. A total of 261 birds of known sex were genotyped and included in this analysis. When known, a birth cohort identifier was specified to minimize the possibility that individuals appear as potential parents of offspring from the same or a previous cohort. Since MasterBayes allows the simultaneous use of genetic and phenotypic data that may inform on parentage, the reconstruction was performed twice: a first time using genotypic information only and a second time including both genetic and morphometric data (including body mass). The genotyping error rates for each locus, the number of unsampled sires and the number of unsampled females were all estimated jointly from the pedigree. Markov chains were run for 1.1 million iterations, with a burn-in of 100 000 iterations and a thinning interval of 1000. Parents (or father or mother) were assigned to an offspring with a 95% joint posterior probability threshold.

### 2.5. RAD-sequencing using pooled DNA samples

To identify loci associated with colour, we used a paired-end RAD-sequencing protocol, using a dataset described previously in which six pools of 18–25 individuals representing the two colour morphs in each of three separate populations (named ‘Bois Ozoux’, ‘Pas de Bellecombe’ and ‘Tévelave’) were sequenced [40]. We took advantage of the recent sequencing of the *Z. lateralis* genome [56] to map reads back onto this reference with BWA MEM (v. 0.7.12) [57], instead of creating consensuses directly from data as in [40]. We aligned contigs and scaffolds from *Z. lateralis* on the zebra finch genome (version July 2008, assembly WUGSC v. 3.2.4) using LASTZ [58]. We removed PCR duplicates using samtools [59]. SNPs were called using Popoolation2 (v. 1.201) [60].

### 2.6. Genotyping by sequencing using individual DNA samples

We further validated the results obtained by the pooled RAD-seq approach by performing a genotyping by sequencing analysis [42] on 42 individuals from the same populations that were used to build the pooled DNA samples (seven individuals per morph per population for a total of 14 individuals in each population). Approximately 1 µg of DNA was extracted with a QIAGEN Blood and Tissue kit following the manufacturer's instructions and sent to the BRC Genomic Diversity Facility at Cornell University [42]. Reads were trimmed with Trimmomatic (v0.33) and mapped on *Z. lateralis* genome using BWA. SNPs were called using freebayes (v0.9.15-1) and filtered with VCFTOOLS (0.1.12b) using the following criteria: (i) a mean sequencing depth between 6 and 20×; (ii) a minimal genotype quality of 20; (iii) a minor allele count of 3, which resulted in around 25 000 unambiguous SNPs.

### 2.7. Genetic structure and association analysis

To validate the lack of genetic structure according to morphs, we performed a PCA [61], using custom scripts in R (v3.2.2) for allele frequencies obtained from pooled data, and the Bioconductor package SeqVarTools for GBS data [62]. Given our sampling strategy, we expected morphs to group together in the PCA and population structure, if any, to correlate with geography. To further rule out the possibility of any neutral structure according to colour morph, we also conducted a locus by locus analysis of molecular variance (AMOVA) in Arlequin v. 3.5 [63] on the 10 115 autosomal SNPs with less than 95% of missing data.

To detect loci displaying a significant association with coloration, we performed an association analysis on the pooled RAD-seq data using the software BAYPASS (v. 2.1) [64]. We computed the empirical Bayesian *p*-value (eBPis) and Bayes factors (BF) expressed in deciban units (dB) to determine the level of association of each SNP with the grey/brown trait. Decibans are a commonly used statistic that describes here the probability that data are produced under a given model. They are often used as a unit for BF. Here, it describes the odds of association to morphs versus the null hypothesis of non-association. BAYPASS was run using default parameters under the core model. Empirical Bayesian *p*-values and BF were then computed for a pseudo-observed dataset containing 1 000 000 SNPs simulated from the actual data. We then compared the observed values to this distribution in order to calibrate the statistics.

For GBS data, we performed a single analysis in PLINK [65] by correcting for population structure with a Cochran–Mantel–Haenszel test (option –mh in PLINK).

### 2.8. Whole-genome sequencing

To validate the hereditary transmission of plumage colour and confirm the validity of pedigrees, 12 individuals from three distinct families were selected for 150 bp paired-end Illumina sequencing. Individuals included 4 brown and 8 grey birds (for reconstructed pedigrees see the electronic supplementary material, table S4). Three types of matings were considered: two grey parents with only grey offspring, two grey parents with both brown and grey offspring, and one parent of each morph with both types of offspring. DNA sequencing libraries were prepared according to Illumina's protocols using the Illumina TruSeq Nano DNA LT Library Prep Kit. Briefly, DNA was fragmented by sonication (Covaris M220) and adaptators were ligated to be sequenced. Eight cycles of PCR were then applied to amplify the libraries. Library quality was assessed using an Agilent Bioanalyzer and libraries were quantified by QPCR using the Kapa Library Quantification Kit. Sequencing was performed in paired-end (2 × 125 pb) on an Illumina HiSeq2500 sequencer at the GeT-PlaGe core facility (INRA, Toulouse). The mean sequencing depth ranged from 6.9× to 11.6× after removing duplicates (9.3× in mean, electronic supplementary material, table S6). After calling with freebayes, SNPs were filtered with VCFTOOLS using the following criteria: (i) a mean sequencing depth between 6 and 20×; (ii) a minimal Phred score of 20 and a genotype quality above 20; (iii) at least eight individuals genotyped.

Putative pedigrees obtained from microsatellite data were confirmed by checking the coefficients of relatedness between individuals in KING (v. 1.4) [66] using 10 000 biallelic SNPs randomly sampled across autosomal scaffolds. An association analysis (including indels) taking into account family structure was performed in LAMP (v. 0.0.12) [67]. We present here results taking into account the putative heredity of the colour trait (brown recessive, grey dominant) as they were similar to those obtained when not taking the transmission mode into account. To assess whether any associated SNP might lead to changes in protein sequence, we performed an annotation using the software SNPdat (v. 1.0.5) [68] and using gene coordinates from [56].

### 2.9. Detecting selective sweeps and characterizing derived and ancestral states

To determine when and on which specific allele selection occurred, we used the coalescent framework implemented in ARGWeaver [69]. ARGWeaver models the coalescent process across non-recombining blocks of sequences and thus provides access to the evolutionary history of DNA sequences. It allows recovering several statistics that describe local genealogies, like coalescence times and local effective population sizes. In the case of a recent and partial selective sweep, selected lineages should display shorter coalescence times than the ancestral ones, i.e. alleles under selection will tend to be younger than neutral alleles.

We ran the analyses using whole-genome sequences from the three parental pairs, as these individuals displayed low levels of relatedness in the whole-genome association analysis and were predicted to display an equal number of brown and grey alleles. To reduce computational burden, we performed the analysis on the scaffold covering the candidate region (scaffold 40) and one on chromosome 2 (scaffold 30) with no SNPs associated with colour. The algorithm was run for 1000 iterations. We then extracted trees and half-time TMRCAs from the output.

## 3. Results

### 3.1. Differences in melanin-based pigmentation patterns between morphs are functionally significant

Brown and grey birds were different in coloration when examined in an avian-appropriate colour space, clustering into two distinct groups along the first component axis in a PCA (figure 1; electronic supplementary material, table S1). All colour variables differed significantly between grey and brown morphs (electronic supplementary material, table S1). In addition, we found that, for all patches, the differences between morphs are greater than the discrimination threshold in the avian visual space (electronic supplementary material, table S1). Taken together, these results highlight the functional significance in relation to colour perception of the differences in pigmentation patterns that distinguish grey and brown birds.

We analysed the melanic content of feathers by determining eumelanin and pheomelanin concentrations (figure 1; electronic supplementary material, table S2). Although levels of eumelanin were similar in both morphs, levels of pheomelanin were clearly lower in the grey morph when compared with the brown morph (ANOVA, *F*_1,20_ = 132.817, *p* = 2.77 × 10^−10^). Pheomelanin was mostly concentrated in feather barbs and rachis (figure 1), suggesting that it is produced and deposited in a timely fashion during feather growth [70]. We also found a highly significant relationship between PC1 scores and the pheomelanin marker (Spearman's *ρ* = 0.834, *p*-value = 7.6 × 10^−6^), consistent with the fact that most of the variation in reflectance spectra between morphs is related to variation in pheomelanin content.

### 3.2. Lack of population structure and Mendelian inheritance reveal a true genetic polymorphism with single-locus control

**Figure 2.**
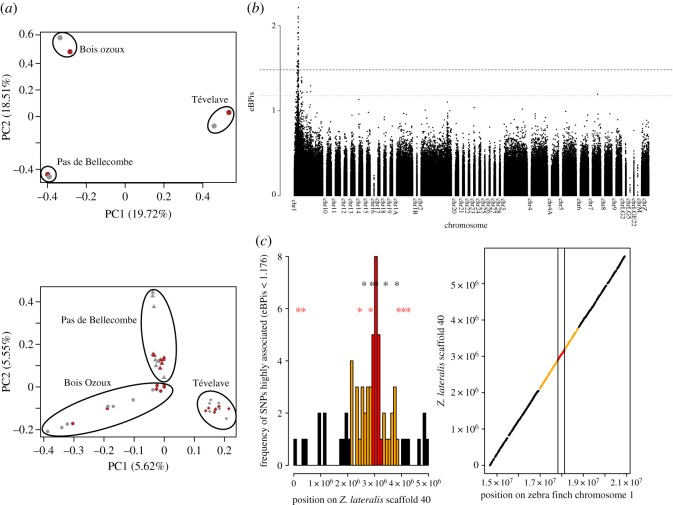
Population structure and association study. (*a*) PCA on allele frequencies for pooled RAD-seq (top) and individual GBS (bottom) data. (*b*) Genome-wide association analysis performed with BAYPASS for 627 795 RAD-seq loci. Horizontal dashed lines correspond to 0.001% (grey) and 0.01 (black) significance thresholds. (*c*) Density of highly associated SNPs on scaffold 40 from the *Zosterops lateralis* reference genome. Upper stars: SNPs displaying both highly significant BF and empirical Bayesian *p*-values (above the 0.001% threshold). Lower stars: 7 GBS SNPs associated with a *p*-value < 1 × 10^−4^. The region including all 100 kb intervals with at least three highly associated SNPs is shown in orange, and the three intervals with the highest density of associated SNPs are shown in red. LASTZ alignment between scaffold 40 and the zebra finch chromosome 1 is also illustrated.

Previous field observations suggested a lack of assortative mating relative to plumage colour in white-eye populations, which should lead to a lack of genetic structure according to morph [30]. Pooled RAD-sequencing and GBS data on 137 and 42 individuals, respectively, from three geographically separate populations revealed that genetic structure was consistent with geography but not with colour morphs. PCA on allele frequencies from pooled RAD-seq data highlighted a clear grouping of brown and grey pools by locality (figure 2*a*). This lack of structure was also found for individual GBS data (figure 2*b*) and was further confirmed by an AMOVA (electronic supplementary material, table S3) which revealed a significant effect of population structure in shaping neutral genetic diversity (*F*_st_ = 0.022, *p*-value < 1 × 10^−5^), yet no effect of colour morph (*F*_ct_ = −0.011, *p*-value = 1).

To study the pattern of inheritance of the two colour morphs, we generated pedigrees for 36 putative parent–offspring triads based on the genotyping of 11 microsatellite markers in a total of 260 individuals. Matings involving two brown parents systematically led to brown offspring, while grey offspring always had at least one grey parent (table 2), suggesting a single-locus control with grey dominant to brown.

**Table 2.** Link between parent and offspring *Zosterops borbonicus* phenotypes. Results were obtained from a pedigree analysis based on 11 microsatellites and morphometric measurements other than colour. Values correspond to pedigree reconstructions congruent between analyses including and excluding morphometric measurements. +1: found in analysis with genetic data only.

parental phenotype	brown offspring	grey offspring
brown × brown	16	0
grey × grey	1	8 (+1)
grey × brown	6	5

### 3.3. Genome-wide association analysis identifies a single genomic region associated with colour phenotype

To identify the genomic regions responsible for the observed colour polymorphism, we performed a genome-wide association analysis on a total of 627 795 SNPs obtained from the pooled RAD-sequencing data, and mapped it onto the *Z. lateralis* genome (figure 2*b*). Thirty-three SNPs had an empirical Bayesian *p*-value (eBPis) higher than 1.48 (at the 0.001% threshold). Among these SNPs, 28 were found on scaffold 40 on the reference genome for *Z. lateralis* which covers approximately positions 15–21 Mb on chromosome 1 of the better assembled zebra finch reference genome (figure 2*c*). Density of strongly associated SNPs (with an eBPis above the 0.01% threshold) was particularly high around 3 Mb of this scaffold, which corresponds approximately to position 18 Mb on the zebra finch chromosome 1. This pattern was further confirmed by individual GBS data, with which seven variants were found to be associated to coloration with an uncorrected *p*-value between 9.1 × 10^−5^ and 8.6 × 10^−7^ on scaffold 40, while only two other variants were found to be associated with similar levels of significance on scaffold 172, which is directly upstream of scaffold 40 according to the zebra finch reference genome.

In sum, we found a single genomic region displaying a clear signal of association with phenotype. Within this region, we identified seven candidate genes based on their known role or on the role of homologues in melanic pigmentation (table 3). Among those genes, four—*AP1S2*, *GPM6B*, *RAB9A* and *TRAPPC2*—lie within regions where associated SNP density is highest (figure 2).

**Table 3.** Summary of seven candidate genes for colour variation in *Zosterops borbonicus*. Information based on OMIM (Online Mendelian Inheritance in Man), a database reporting large-scale genotype–phenotype associations in humans and laboratory mice. Positions correspond to coordinates on zebra finch reference genome.

candidate gene	complete name	position (from first to last exon)	role in melanocyte	impact on phenotype	references
*RS1*	*retinoschisin*	15 288 297–15 291 738	retinal cells adhesion, cell–cell interaction	depigmentation in retinal pigment epithelium	[71]
*AP1S2*	*adaptor-related protein complex 1, sigma 2 subunit*	16 724 833–16 754 005	AP-1 complex is involved in melanosome genesis and is necessary for TYRP1 to reach the melanosome and produce eumelanin	mutations on another complex, AP-3, lead to the pearl (*Ap3b1*; coat hypopigmentation) and mocha (*Ap3d1*; coat colour dilution) phenotypes in mice	[72–74]
*GPM6B*	*glycoprotein M6B*	17 812 158–17 843 054	membrane protein involved in neuronal tissues. Up-regulated by *MITF*	—	[75]
*TRAPPC2*	*trafficking protein particle complex, subunit 2*	17 877 165–17 880 771	TRAPP complex is involved in vesicle transport and tethering	in mice, mutations on the subunit *TRAPPC6A* lead to hypopigmented patches in the coat and retinal epithelium	[76]
*RAB9A*	*Ras-associated protein 9A*	17 886 048–17 886 836	regulation of vesicular trafficking. Interacts with BLOC-3, involved in Hermansky-Pudlak syndrome	*Rab38*: chocolate phenotype in mice. *Rab27a:* Griscelli syndrome (hypomelanosis and neurological defects)	[28,77,78]
*APXL*	*apical protein of Xenopus-like*	19 986 240–20 035 565	melanosome biogenesis and transport. Activity requires the G-protein *RAB27A*	ocular albinism	[79]
*OA1*	*ocular albinism1*	20 136 404–20 149 393	melanosome transport and interactions with cytoskeleton	ocular albinism	[80,81]

### 3.4. Functionally important changes lie outside candidate gene coding regions

**Figure 3.**
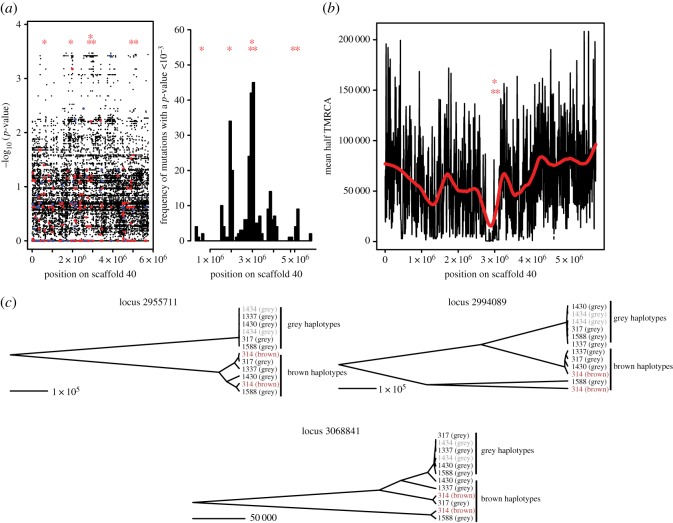
Test for selection using whole-genome sequencing. (*a*) Association analysis of whole-genome sequencing data, including four brown and eight grey individuals from three different families. Synonymous and non-synonymous SNPs are highlighted in blue and red, respectively. Stars indicate the positions of the seven candidate genes listed in table 3. Density of SNPs associated with a *p*-value < 1 × 10^−3^ is also provided. (*b*) Plot of half-TMRCA (in number of generations) for scaffold 40. Stars indicate the position of the three non-recombining blocks examined in (*c*). (*c*) Genealogies observed at three distinct points from the colour locus. All three blocks of sequences included SNPs associated with colour with a *p*-value < 1 × 10^−3^ in the whole-genome association analysis. Branch lengths represent time in generations. Individual phenotype is indicated in brackets. Individuals 1434 (grey morph) and 314 (brown morph) are found to be homozygous at SNPs strongly associated with colour.

Having pinpointed the locus responsible for plumage colour variation, we searched for functional polymorphism at non-synonymous sites in the candidate region by obtaining whole-genome sequences from 12 individuals sampled across three distinct families from different populations (electronic supplementary material, table S4). Coefficients of kinship estimated from whole-genome data for these individuals were consistent with the pedigrees estimated from microsatellite data (electronic supplementary material, table S5). The highest density of associated SNPs was found between 17.7 and 18.7 Mb on the zebra finch chromosome 1 (figure 3*a*). Only one non-synonymous substitution at a level of significance less than 1 × 10^−3^ was found in *ACE2*, an angiotensin (figure 3*a*). The induced change led to the substitution of methionin to isoleucin, two hydrophobic amino acids which are extremely similar. Therefore, there is no non-synonymous change that would make a suitable candidate for the observed phenotypic variation. This suggests a role for regulatory mutations in determining the observed phenotype.

### 3.5. Evidence for a selective sweep suggests recent selection on the grey allele

To assess whether selection had a role in the emergence of the observed polymorphism and to identify which of the two allele categories (brown or grey) is ancestral, we sampled ancestral recombination graphs for the entire candidate scaffold. Here, we took advantage of the fact that the individuals used in this test displayed six ‘brown’ and six ‘grey’ alleles at the most strongly associated markers. We found that the candidate region displayed a clear signature of a recent selective sweep, with an extremely short half-TMRCA between positions 2 700 000 and 3 100 000 on scaffold 40 (figure 3*b*). An examination of the local trees for regions containing SNPs associated with phenotype clearly showed that the most recent haplotypes were found in grey individuals only (figure 3*c*). These haplotypes also harboured the allele associated with grey coloration in the association analysis. Median half-TMRCA was estimated at 1733 generations in the region, significantly shorter than the estimate for the remaining regions included in the analysis (median: 54 460 generations; Wilcoxon rank test, *p* < 2.2 × 10^−16^). This indicates that grey coloration has been positively selected and that brown and grey phenotypes represent ancestral and derived conditions, respectively.

## 4. Discussion

We confirmed that highland populations of the Reunion grey white-eye present a genetic colour polymorphism that results from the differential deposition of pheomelanin on feathers, leading to predominantly grey or brown plumage, and is controlled by a simple genetic mechanism with a recessive ‘brown’ allele and a dominant ‘grey’ allele. A genome-wide association analysis designed to search for loci involved in the polymorphism led to the identification of a single genomic region on chromosome 1 characterized by SNPs strongly associated with colour phenotype. A coalescent analysis of selection pinpoints the same region, and suggests recent selection on the grey allele. Annotation of these SNPs using the *Z. lateralis* reference genome [56] did not reveal any non-synonymous mutations or deletions in coding parts of the genomic region associated with variation between colour morphs, indicating that functionally important nucleotide changes in the Reunion grey white-eye must lie outside coding regions. This suggests a role for *cis*-regulatory mutations that either prevent the synthesis of pheomelanin in grey feathers, or increase its production in brown ones. The first mechanism seems more likely in the present case since the grey phenotype seems to represent the derived condition. The fact that eumelanin is found in both brown and grey feathers, while pheomelanin is only present in brown feathers suggests that the underlying genes may be involved in melanogenesis and melanosome transport.

Importantly, the genomic scaffold associated with plumage colour does not include, to the best of our knowledge, genes previously known to be involved in hair or feather coloration in other species, even in model species such as laboratory mice. Three genes found in this region, *RS1*, *APXL* and *OA1*, are known to affect melanogenesis and pigmentation in retinal pigment cells. In addition to these genes, the colour locus includes genes (*GPM6B*, *RAB9A*, *TRAPPC2*, *AP1S2*) known to be involved in intracellular trafficking and in shaping melanosomes in melanocytes (table 3). Among them, *GPM6B* is of particular interest as it is regulated by *MITF*, a key transcription factor involved in melanocyte specification [75]. However, while changes in *GPM6B* expression were found in a comparison of black and hooded crows [30], there is currently no direct evidence for a role of this gene in feather coloration. Our results also confirm previous findings suggesting that variation at genes often studied in natural populations, such as *MC1R*, *Agouti* or *POMC* [32,82,83], is not directly related to colour variation in the Reunion grey white-eye [38] and provide an impressive illustration of the diversity of the mechanisms underlying melanin-based plumage colour evolution in birds and perhaps other vertebrates. Future work should focus on gene expression differentiation to define the developmental basis of this colour polymorphism and to determine, for example, if the candidate loci found on chromosome 1 are epistatic over other genes classically found associated with melanin-based colour variation.

A large-effect locus can break up into many small- to moderate-effect mutations [32,84]. Thus, although the pattern of inheritance of plumage colour and the existence of a single strongly associated genomic region points towards a relatively simple genetic mechanism, it remains possible that several of the candidate genes we identified are simultaneously involved in shaping the observed colour variation in this study. If mutations arose independently at several genes involved in melanogenesis, their physical proximity would favour linkage between the best combinations of mutations by sieve. Structural rearrangements such as large inversions covering the genomic region associated to colour, if any, would also stabilize associations between several alleles [85]. In that case, the colour locus that we identified in this study may be a further example of the so-called ‘largesse of the genome’ [86,87]—i.e. the predisposition of certain genomic regions to mediate integrated phenotypic shifts—in vertebrates.

Genes like *RS1*, *APXL* or *OA1* have been studied in the context of ocular diseases or melanomas [88], in a way that suggests important functions in melanocyte development (table 3). Therefore, they probably play a role in ocular development and in colour recognition, which might have an impact on how individuals recognize and favour conspecifics. In addition, *GPM6B* is known to be involved in neural development, serotonin uptake and bone formation [89,90]. Thus, this gene could influence feather colour, brain development and behaviour. This feature is particularly interesting, since changes in behavioural strategies associated with visual signalling can explain the persistence of morphs over time [91].

Our work reveals a new genomic region not previously associated with melanic coloration in vertebrates, and underscores the importance of genome-based research on non-model species to understand the genetic basis of ecologically significant traits and their role in phenotypic diversification [92]. Our results suggest a strong selective advantage for the dominant ‘grey’ allele once it arose from a new mutation, leading to its fast spread across all highland populations of the Reunion grey white-eye [93]. Similar patterns of selection on *de novo* mutations at colour genes have been recently described in deer mice (*Peromyscus maniculatus)*, where multiple independent mutations were selected for cryptic coloration after the colonization of a novel selective environment [31,32]. In white-eyes, both colour morphs are found at an appreciable frequency in all populations, implying that the selected allele does not go to fixation, but instead reaches an intermediate frequency, as would be expected under balancing selection. This may happen as the result of, for example, heterozygote advantage or negative frequency-dependent selection [94]. Distinguishing between these possibilities is notoriously difficult, and future studies should estimate the relative survival and fertility rates of the different ‘colour’ genotypes in order to determine the nature of selection acting on this colour polymorphism and to come closer to an explanation about its persistence.

## Supplementary Material

Supplementary Methods and TablesThis file includes details about methods and parameters used, as well as supplementary results on analyses ofphenotype and whole genome analyses.

## Data accessibility

All data are deposited on Dryad (phenotypic data, GBS, http://dx.doi.org/10.5061/dryad.1q9g7) [95] and European Nucleotide Archive repositories (raw reads for whole genomes and pooled RAD-seq, accession nos. ERP020509 and ERP002555). Methods and options are further detailed in the electronic supplementary material. Scripts used in this study are freely available on GitHub at https://github.com/YannBourgeois/Scripts_Pool_RADseq.
