# Supplementary material for: A novel locus on chromosome 1 underlies the evolution of a melanic plumage polymorphism in a wild songbird
Source: R Soc Open Sci. 2017 Feb 15;4(2):160805. doi: 10.1098/rsos.160805 (PMC5367300; doi:10.1098/rsos.160805)
Supplement: Supplementary Methods and TablesThis file includes details about methods and parameters used, as well as supplementary results on analyses ofphenotype and whole genome analyses. [file rsos160805supp1.docx]

Supplementary Methods

**Measurement of the reflectance spectra of plumage patches**

Spectral data were obtained in the laboratory with a USB 2000 spectrophotometer connected to a PX-2 light source via a Qt-200 bifurcate optical fibre probe (Ocean Optics, Dunedin, FL, USA). We tested for differences among variants in each individual morphological trait and in each plumage patch using non-parametric ANOVAs. Determining whether birds are actually able to discriminate between color morphs is necessary to confirm the biological significance of plumage color variation. We used Vorobyev & Osorio’s [1] model of color perception to calculate ΔS, the chromatic contrast between two colors, in units of just-noticeable-differences (JNDs), where 1 JND is the threshold value of discrimination between two colors.

**Pooled RAD-sequencing**

We pooled individuals per color and locality, and the six sequenced pools included between 18 and 25 individuals, totaling 137 individuals, with distinct barcodes discriminating morphs and localities (but not individuals). Sequencing was performed on an Illumina HiSeq 2000.

**RAD-sequencing**

SNPs were called using the tools from Popoolation2 (v1.201) [2]. We used the following stringency criteria: a minimal sequencing depth of 10X by library, a minimal allele count of 2 in the whole dataset and a minimal quality (Phred) score of 20.

**Genome-wide association analysis**

BAYPASS (v2.1) [3] is an elaboration on the BAYENV model [4,5] that estimates and explicitly takes into account the hierarchical structure of populations to detect signatures of local adaptation from variation in allele frequencies. We computed the empirical Bayesian p-value (eBPis) and Bayes factors (BF) in decibans to determine the level of association of each SNP with the gray/brown trait. BAYPASS was run using default parameters under the core model: MCMC chains were run for 25,000 iterations after a 5,000 iterations burn-in period. Samples were taken from the chain every 25 iterations. We simulated 1,000,000 SNPs under the demographic model inferred from the actual dataset by BAYPASS using the R function simulate.baypass() provided with the software. eBPis and BF were then computed for this pseudo-observed dataset (POD) to determine their distribution under a neutral model. We then compared the observed values to this distribution in order to calibrate the statistics.

**ARGWeaver**

We used only whole-genome data for the six parents from the three families used in whole genome sequencing to limit biases due to relatedness. Haplotypes from the six parental whole genomes were phased together using SHAPEIT2v2.r790 [6] with default parameters for each scaffold. We then ran ARGWeaver using a mutation rate of 3.6.10^-9^ mutations per year [7], a recombination rate of 1.5.10^-8^ events per year [8], a compression of 10bp for the sequences, a maximum time for coalescence of one million generations and an effective population size of 100,000 individuals, consistent with previous census [9] and genetic estimates [10].

Supplementary Tables

Table S1

Measures of color disparity were calculated for each of the four plumage patches considered and each pair of individuals. The within- and among-variant averages are given, showing for which patches and which variables the plumage of *Z. borbonicus* is more different among than within morphs. Color span in JNDs indicates to what extent birds are able to discriminate between pairs of colors. When color span is greater than 1 JND (underlined), the difference is perceivable to the bird’s eye. npANOVA tests for difference between morphs are provided with their p-values.

| **Back** | | | **npANOVA** | |
| --- | --- | --- | --- | --- |
|  | Average within-morph | Average between morph | R² | P |
| Normalized brillance disparity (%) | 5.91 x 10^-3^ | 6.07 x 10^-3^ | 0.03 | 0.12 |
| Achieved chroma disparity (%) | 7.36 x 10^-2^ | 2.23 x 10^-1^ | 0.75 | < 0.001 |
| Hue disparity (radian) | 1.01 x 10^-1^ | 2.08 x 10^-1^ | 0.51 | < 0.001 |
| Colour span (Euclidean distance) | 2.48 x 10^-2^ | 8.93 x 10^-2^ | 0.83 | < 0.001 |
| Colour span (bird JND) | 1.73 | 6.15 | 0.82 | < 0.001 |
|  |  |  |  |  |
|  |  |  |  |  |
| **Head** | | | **npANOVA** | |
|  | Average within-morph | Average between morph | R² | P |
| Normalized brillance disparity (%) | 7.29 x 10^-3^ | 7.72 x 10^-3^ | 0.07 | 0.009 |
| Achieved chroma disparity (%) | 8.25 x 10^-2^ | 2.16 x 10^-1^ | 0.70 | < 0.001 |
| Hue disparity (radian) | 1.06 x 10^-1^ | 1.77 x 10^-1^ | 0.38 | < 0.001 |
| Colour span (Euclidean distance) | 2.66 x 10^-2^ | 8.52 x 10^-2^ | 0.79 | < 0.001 |
| Colour span (bird JND) | 1.86 | 5.91 | 0.77 | < 0.001 |
|  |  |  |  |  |
|  |  |  |  |  |
| **Flank** | | | **npANOVA** | |
|  | Average within-morph | Average between morph | R² | P |
| Normalized brillance disparity (%) | 1.32 x 10^-2^ | 1.77 x 10^-2^ | 0.27 | < 0.001 |
| Achieved chroma disparity (%) | 1.07 x 10^-1^ | 2.11 x 10^-1^ | 0.49 | < 0.001 |
| Hue disparity (radian) | 8.76 x 10^-2^ | 2.08 x 10^-1^ | 0.61 | < 0.001 |
| Colour span (Euclidean distance) | 4.13 x 10^-2^ | 9.77 x 10^-2^ | 0.61 | < 0.001 |
| Colour span (bird JND) | 3.07 | 7.09 | 0.61 | < 0.001 |
|  |  |  |  |  |
|  |  |  |  |  |
| **Belly** | | | **npANOVA** | |
|  | Average within-morph | Average between morph | R² | P |
| Normalized brillance disparity (%) | 3.09 x 10^-2^ | 3.07 x 10^-2^ | 0.00 | 0.810 |
| Achieved chroma disparity (%) | 6.56 x 10^-2^ | 7.02 x 10^-2^ | 0.07 | 0.021 |
| Hue disparity (radian) | 2.53 x 10^-1^ | 2.64 x 10^-1^ | 0.07 | 0.011 |
| Colour span (Euclidean distance) | 2.01 x 10^-2^ | 2.12 x 10^-2^ | 0.06 | 0.022 |
| Colour span (bird JND) | 1.22 | 1.28 | 0.06 | 0.031 |

Table S2

Summary of the different ANOVAs performed on different types of melanin content across brown and gray individuals.

|  | Morph | Sex | Morph*Sex |
| --- | --- | --- | --- |
| Total melanin content | F_1,20_ = 0.879, P = 0.359 | **F_1,20_ = 7.578,P = 0.012*** | F_1,20_ = 0.745**,P**= 0.398 |
| Eumelanin content | F_1,20_ = 0.268, P = 0.610 | F_1,20_ = 1.113**,P**= 0.304 | F_1,20_ = 3.063**,P**= 0.095 |
| Pheomelanin content | **F_1,20_ = 132.817, P = 2.77*10^-10^ ***** | F_1,20_ = 1.225**,P**= 0.281 | F_1,20_ = 0.927**,P**= 0.347 |

Table S3

Locus-by-locus molecular analysis of variance (AMOVA) performed on 42 individuals (21 brown and 21 gray) at 10,115 high-quality SNP loci from GBS.

| Source of variation | Percentage of variance | | F-statistics | Significance |
| --- | --- | --- | --- | --- |
| Among morphs | | -1.11 | -0.011 | 1 |
| Among localities within morphs | | 3.35 | 0.033 | < 1.10^-5^ |
| Within localities | | 97.76 | 0.022 | < 1.10^-5^ |

Table S4. Putative pedigrees for the twelve birds selected for whole-genome sequencing.

| **Identifier** | **Morph** | **Family** | **Status** | **Sex** |
| --- | --- | --- | --- | --- |
| 314 | BROWN | 1 | parent | Male |
| 317 | GRAY | 1 | parent | Female |
| 320 | BROWN | 1 | offspring | Female |
| 11-869 | BROWN | 1 | offspring | Male |
| 319 | GRAY | 1 | offspring | Female |
| 1430 | GRAY | 2 | parent | Male |
| 1434 | GRAY | 2 | parent | Female |
| 1685 | GRAY | 2 | offspring | Female |
| 1708 | GRAY | 2 | offspring | Female |
| 1337 | GRAY | 3 | parent | Male |
| 1588 | GRAY | 3 | parent | Female |
| 1579 | BROWN | 3 | offspring | Male |

Table S5. Kinship coefficients matrix for the 12 individuals used in WGS. Relatedness was calculated in KING on the basis of 10,000 random autosomal SNPs. In bold are indicated pairs for which kinship coefficient was higher than expected. This occurred only between parents, suggesting partial inbreeding in the studied populations.

| Family | Individual 1 | Individual 2 | Expected kinship | Observed kinship |
| --- | --- | --- | --- | --- |
| F1 | 11-869 | 314 | 0.25 | 0.252 |
| F1 | 11-869 | 317 | 0.25 | 0.2256 |
| F1 | 11-869 | 319 | 0.25 | 0.1935 |
| F1 | 11-869 | 320 | 0.25 | 0.2522 |
| **F1** | **314** | **317** | **0** | **0.0681** |
| F1 | 314 | 319 | 0.25 | 0.2488 |
| F1 | 314 | 320 | 0.25 | 0.2399 |
| F1 | 317 | 319 | 0.25 | 0.3051 |
| F1 | 317 | 320 | 0.25 | 0.3184 |
| F1 | 319 | 320 | 0.25 | 0.2886 |
| **F2** | **1430** | **1434** | **0** | **0.0745** |
| F2 | 1430 | 1685 | 0.25 | 0.2439 |
| F2 | 1430 | 1708 | 0.25 | 0.2417 |
| F2 | 1434 | 1685 | 0.25 | 0.3104 |
| F2 | 1434 | 1708 | 0.25 | 0.3118 |
| F2 | 1685 | 1708 | 0.25 | 0.2795 |
| F3 | 1337 | 1579 | 0.25 | 0.286 |
| **F3** | **1337** | **1588** | **0** | **0.0671** |
| F3 | 1579 | 1588 | 0.25 | 0.2409 |

Table S6. Mean depth of coverage for whole-genomes after duplicates removal (given an assembled reference genome of 1.036 Gb)

| **Individual** | **Number of reads mapped** | **Mean depth** |
| --- | --- | --- |
| 319 | 57130148 | 6.9 |
| 1588 | 58107165 | 7.0 |
| 1708 | 66646148 | 8.0 |
| 11-869 | 72241490 | 8.7 |
| 1685 | 73490335 | 8.9 |
| 1337 | 78001866 | 9.4 |
| 1434 | 79459085 | 9.6 |
| 314 | 83393649 | 10.1 |
| 320 | 84005318 | 10.1 |
| 1579 | 86150759 | 10.4 |
| 317 | 89835475 | 10.8 |
| 1430 | 95765452 | 11.6 |

References

1. Vorobyev, M. & Osorio, D. 1998 Receptor noise as a determinant of colour thresholds. *Proc. Biol. Sci.* **265**, 351–8. (doi:10.1098/rspb.1998.0302)

2. Kofler, R., Pandey, R. V. & Schlötterer, C. 2011 PoPoolation2: identifying differentiation between populations using sequencing of pooled DNA samples (Pool-Seq). *Bioinformatics* **27**, 3435–6. (doi:10.1093/bioinformatics/btr589)

3. Gautier, M. 2015 Genome-Wide Scan for Adaptive Divergence and Association with Population-Specific Covariates. *Genetics* **201**, 1555–1579.

4. Coop, G., Witonsky, D., Di Rienzo, A. & Pritchard, J. K. 2010 Using environmental correlations to identify loci underlying local adaptation. *Genetics* **185**, 1411–1423. (doi:10.1534/genetics.110.114819)

5. Günther, T. & Coop, G. 2013 Robust identification of local adaptation from allele frequencies. *Genetics* **195**, 205–220. (doi:10.1534/genetics.113.152462)

6. O’Connell, J. et al. 2014 A General Approach for Haplotype Phasing across the Full Spectrum of Relatedness. *PLoS Genet.* **10**. (doi:10.1371/journal.pgen.1004234)

7. Axelsson, E., Smith, N. G. C., Sundström, H., Berlin, S. & Ellegren, H. 2004 Male-biased mutation rate and divergence in autosomal, z-linked and w-linked introns of chicken and Turkey. *Mol. Biol. Evol.* **21**, 1538–47. (doi:10.1093/molbev/msh157)

8. Backström, N. et al. 2010 The recombination landscape of the zebra finch *Taeniopygia guttata* genome. *Genome Res.* **20**, 485–95. (doi:10.1101/gr.101410.109)

9. Bertrand, J. A. M., Bourgeois, Y. X. C. & Thébaud, C. 2015 Population density of the Réunion Grey White- eye Zosterops borbonicus within the summit ecosystems of Réunion , Mascarene Islands. **6525**, 0–4. (doi:10.2989/00306525.2015.1080198)

10. Bourgeois, Y. X. C. 2013 Génétique évolutive d’un cas extrême de polymorphisme de la coloration du plumage chez un oiseau insulaire, Zosterops borbonicus (Zosteropidae).
